# Quercetin Improves Cognitive Function by Ameliorating Histopathological Changes and Inflammation in Di(2-ethylhexyl) Phthalate-Exposed Mice

**DOI:** 10.3390/brainsci16040431

**Published:** 2026-04-20

**Authors:** Leila Nadalinezhad, Maryam Ghasemi-Kasman, Mohsen Pourghasem, Fatemeh Rabiei, Farideh Feizi, Farzin Sadeghi

**Affiliations:** 1 Student Research Committee, Babol University of Medical Sciences, Babol 47176-47745, Iran; nadalinezhadl56@yahoo.com (L.N.); fatemehrabieibahar@gmail.com (F.R.); 2 Department of Anatomy, Embryology and Histology, Faculty of Medicine, Babol University of Medical Sciences, Babol 47176-47745, Iran; mpourghasem@hotmail.com (M.P.); faridehfeizi@yahoo.com (F.F.); 3 Cellular and Molecular Biology Research Center, Health Research Institute, Babol University of Medical Sciences, Babol 47176-47745, Iran; sadeghifarzin6@gmail.com; 4 Department of Physiology, Faculty of Medicine, Babol University of Medical Sciences, Babol 47176-47745, Iran; 5 Department of Medical Virology and Biotechnology, Faculty of Medicine, Babol University of Medical Sciences, Babol 47176-47745, Iran

**Keywords:** DEHP, spatial memory, hippocampus, quercetin, inflammation

## Abstract

**Highlights:**

**What are the main findings?**

- Quercetin reduced Di(2-ethylhexyl) phthalate-induced upregulation of inflammatory genes and preserved neuronal structure.
- Quercetin improved cognitive function and attenuated hippocampal astrocyte activation in Di(2-ethylhexyl) phthalate exposed mice.

**What are the implications of the main findings?**

- Quercetin improves cognitive function via attenuating inflammation and neuronal loss.
- Quercetin as a natural product may have therapeutic potential to mitigate Di(2-ethylhexyl) phthalate-induced neurotoxicity.

**Abstract:**

**Background/Objectives**: Phthalates are a group of organic compounds widely used for enhancement in flexibility and transparency of polyvinyl chloride (PVC) products. Exposure to phthalate-containing substances has been shown to affect brain function, particularly in learning and memory processes. Quercetin is a plant-derived flavonoid with remarkable anti-oxidant and anti-inflammatory potential. This study investigated the possible protective effects of quercetin on spatial learning and memory, histomorphometric changes, and hippocampal expression of inflammatory cytokines (*TNF-α* and *IL-6*) in male mice exposed to di(2-ethylhexyl) phthalate (DEHP). **Methods**: A total of 42 male mice were divided into seven groups. Quercetin was administered orally at doses of 25 and 50 mg/kg/day, either alone or in combination with DEHP (200 mg/kg/day). Following the final day of the treatment, spatial learning and memory were assessed by the Morris Water Maze test. Hippocampal tissues were sampled for Nissl, H&E, and immunofluorescence staining. Quantitative real-time PCR was used to measure the expression of *TNF-α* and *IL-6*. **Results**: The DEHP group exhibited significant impairments in learning and memory, neuronal damage, and cellular disorganization in the hippocampus, along with increased astrocyte activation and elevated expression of *TNF-α* and *IL-6*. On the other hand, quercetin supplementation significantly reduced these inflammatory markers and histological damages and also improved spatial learning and memory. **Conclusions**: Overall, quercetin improves cognitive function that is associated with attenuating astrocyte activation and inflammation.

## 1. Introduction

Phthalates are a class of synthetized organic chemicals which are used mostly to enhance the flexibility, durability, and transparency of polyvinyl chloride (PVC) and also other polymer-based products [1]. Among them, di(2-ethylhexyl) phthalate (DEHP) is one of the most commonly used compounds, found in a wide variety of consumer services, including food packaging, cosmetics, toys, and specifically medical devices [2,3]. Due to their non-covalent bond to polymer materials, phthalates are expected to result in widespread human exposure through various ways such as ingestion, inhalation, dermal absorption, and medical interventions [4].

Rising evidence suggests that DEHP may biologically exert various toxic effects on different organ systems, including the endocrine [5], reproductive [6], hepatic [7], and also nervous systems [8]. Its neurotoxic potential has been growing some concerns, particularly during early development. DEHP has been shown to cross the blood–brain barrier (BBB) and impair neuronal function by inducing oxidative stress, mitochondrial dysfunction, and neuroinflammation, all of which disrupts synaptic plasticity and leads to cognitive deficits [2,9]. In experimental studies, DEHP exposure has been associated with decreased spatial memory, reduced neuronal intensity, and elevated levels of pro-inflammatory cytokines such as tumor necrosis factor-alpha (TNF-α) and interleukin-6 (IL-6) in the hippocampus region [10,11]. Oxidative stress and inflammation are key mediators of DEHP-induced neuronal loss. Excessive generation of reactive oxygen species (ROS) leads to lipid peroxidation, DNA damage, and protein oxidation, causing cell death eventually. Additionally, activation of astrocytes and microglia induces the release of inflammatory mediators, further worsening neuronal damage [12].

Due to the limitations of conventional pharmacological treatments in addressing environmental neurotoxins, there is a rising attention toward naturally occurring compounds with antioxidant and anti-inflammatory potentials. Quercetin, a flavonoid found vastly in various fruits and vegetables such as apples, onions, and berries, has been investigated for its neuroprotective capabilities [13,14]. Quercetin plays its role as a free radical scavenger, modulates intracellular signaling pathways (e.g., NF-κB), and reduces the expression of TNF-α and IL-6 [13,15]. Quercetin has also been reported to improve learning and memory performance in experimental models of neurotoxicity, ischemia, and neurodegeneration [16].

Despite the rising attention of DEHP’s impact on the nervous system, there is a lack of knowledge regarding the potential protective effects of quercetin against DEHP-induced cognitive and structural dysfunctions in the brain. Therefore, the present study aimed to investigate whether quercetin could inhibit the negative effects of DEHP on spatial learning and memory, hippocampal histomorphology, astrocyte activation, and expression of inflammatory markers in male mice.

## 2. Materials and Methods

### 2.1. Chemicals

Di-(2-ethylhexyl) phthalate (DEHP, 99% purity) and quercetin (99% purity) were obtained from Merck (Darmstadt, Germany) and Sigma-Aldrich (Darmstadt, Germany), respectively.

### 2.2. Animals

A total of 42 adult male NMRI mice (8–10 weeks, 25–35 g) were provided from the Animal Facility of Babol University of Medical Sciences (Babol, Iran). The mice were housed in standard polypropylene cages, under controlled environmental conditions, a temperature of 22 ± 2 °C, relative humidity of 55 ± 5%, and a 12 h light/dark cycle. They had ad libitum access to food and water. The study protocol was approved by the Ethics Committee of Babol University of Medical Sciences (Approval No. IR.MUBABOL.HRI.REC.1398.192). All animal experiments were complied with the ARRIVE guidelines and carried out in accordance with the U.K. Animals (Scientific Procedures) Act, 1986 and associated guidelines, EU Directive 2010/63/EU for animal experiments, or the National Research Council’s Guide for the Care and Use of Laboratory Animals.

### 2.3. Experimental Groups

**Figure 1:**
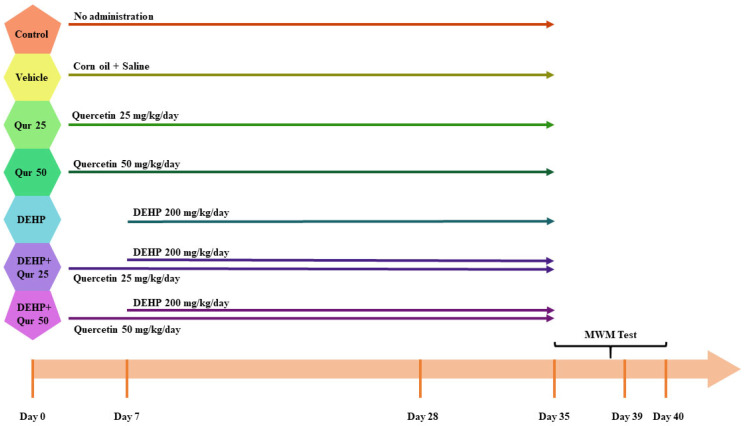
Timing of induction, treatment, and behavioral measurement.

The mice were randomly assigned into seven groups (n = 6). The groups were as follows: the control group, which received no treatment; the vehicle group, which was administered corn oil and saline for five weeks [2,17,18,19]; the quercetin 25 group, which received 25 mg/kg/day of quercetin orally for five weeks [20]; the quercetin 50 group, treated with 50 mg/kg/day of quercetin orally for five weeks [18,20]; the DEHP group, which was given 200 mg/kg/day of DEHP orally for four weeks [21]; the DEHP + quercetin 25 group, which received 25 mg/kg/day of quercetin starting one week before and continuing during the four weeks of DEHP exposure [20]; and the DEHP + quercetin 50 group, administered 50 mg/kg/day of quercetin following the same treatment protocol. It is worth to mention that the DEHP dose (200 mg/kg/day, oral, 4 weeks) was selected based on the previously cited rodents’ studies representing that 100–500 mg/kg/day range demonstrates oxidative stress, inflammatory response initiation, and histopathological changes within 3–6 weeks. Also, the 4-week duration was chosen to capture sub-chronic exposure that more clearly avoids additional confounding factors such as aging-related alterations and to remain with published protocols introduced. The timing of induction, treatment and behavioral measurement is represented as Figure 1 for better clarity.

It also should be mentioned that throughout the different procedures and assays of this article, sample sizes are inspected differently due to the nature of experimental design and the exclusive outcomes based on the different questions being addressed. However, “n” represents the number of individual mice per groups in all assays including MWM, histological and IF analyses. No animals were excluded from any experiment. All data gathered from each animal were therefore in the analyses. Additionally, to ensure that fields/sections are not considered as individual units, all statistical analyses state for the animal as the primary experimental unit.

### 2.4. Morris Water Maze (MWM)

Following the final day of treatment, spatial learning and memory performance were evaluated using the MWM over a period of five consecutive days [22]. The testing pool was a circular tank with 120 cm of diameter filled with water which was kept at a temperature of 24 °C. A circular pistol was centrally placed within the pool. Mice were tested for 5 days, 4 days of acquisition trials followed with a single probe trial. During the first four days, each mouse performed 4 trials per day, initiating from four different randomized start positions around the pool perimeter with the maximum trial duration of 90 s. Additionally, 60 s inter-trial interval was performed, during which mice were allowed to rest on the platform. On the probe trial (fifth day), the platform is hence removed, and the mouse was allowed to swim freely for 60 s. Data gathered from four-day trials were averaged to examine learning performances. Also, probe trial data were analyzed. The time taken to locate the hidden platform (escape latency), swim path length, swimming speed, and time spent in the target quadrant were recorded and analyzed using EthoVision^®^ XT 11.5 software (Noldus Information Technology B.V., Wageningen, The Netherlands).

### 2.5. Brain Tissue Collection

The mice were anesthetized with an intraperitoneal injection of chloral hydrate at a dose of 300 mg/kg. The right hippocampus was rapidly dissected and stored at −80 °C for subsequent RNA extraction and quantitative real-time PCR (qRT-PCR) analysis. The left hippocampus was divided into two parts as follows: one part was fixed in 4% paraformaldehyde and embedded in paraffin for histological staining, including Nissl [23] and hematoxylin-eosin (H&E) [24]; the other part was embedded in optimal cutting temperature (OCT) and instantly used for immunofluorescence staining.

### 2.6. Histological Evaluation

Sections of paraffin-embedded tissue, each 6 µm thick, were prepared and stained with 0.1% Cresyl violet for Nissl staining in the CA1 and CA3 regions of the hippocampus. H&E staining was used to evaluate general cellular morphology. The stained sections were examined under an Olympus BX51 light microscope (Olympus Corporation, Tokyo, Japan). To minimize sampling bias, neuronal cells were counted manually in three randomly selected microscopic fields per section using by an observer blinded to the treatment groups [25]. Three non-overlapping regions of interest (ROIs) of 400 μm × 400 μm were selected per section using ImageJ software 1.42 V (National Institutes of Health, Bethesda, MD, USA). The ROIs were located systemically, ensuring similar spacing and edge effect avoidance. Neurons were manually counted within each ROI. Damaged or pyknotic neurons were marked based on the following criteria: (1) condensed, darkly stained chromatin clumped against the nuclear membrane stated as pyknosis, (2) fragmented or shrunken nuclei, and (3) cellular shrinkage and increased eosinophilia in the cytoplasm. Only cells meeting this checklist were considered as dark/pyknotic cells.

### 2.7. Immunofluorescence Staining

Cryosections, 6 µm thick, were blocked with 10% normal goat serum to prevent non-specific bindings. The sections were incubated overnight at 4 °C with a rabbit anti-GFAP primary antibody (1:400, Z0334, Dako, Glostrup, Denmark). The following day, they were incubated with a goat anti-rabbit IgG Alexa Fluor^®^488 secondary antibody (1:1000, ab150077, Abcam, Cambridge, UK). Nuclei were counterstained using 4′,6-diamidino-2-phenylindole (DAPI). Slides were examined under an Olympus IX71 fluorescence microscope, and GFAP-positive cells were counted in the CA1 and CA3 regions in three non-overlapping fields per section [25].

### 2.8. qRT-PCR

Total RNA was extracted from the right hippocampus using the ParsTous RNA extraction kit (Mashhad, Iran) according to the manufacturer’s protocol. Complementary DNA (cDNA) was synthesized from the extracted RNA using a commercial reverse transcription kit provided by ParsTous (Mashhad, Iran). The expression levels of target genes, *TNF-α* and *IL-6*, were normalized to *GAPDH* as the house-keeping gene. The relative expression was calculated using the 2^−ΔΔCt^ method [26]. The primer sequences used in this study are shown in Table 1.

### 2.9. Statistical Analysis

All quantitative data were expressed as mean ± standard error of the mean (SEM). The normality of data distribution was assessed using the Shapiro–Wilk test. For the MWM data across the four days of training, repeated-measure ANOVA was employed to analyze the effect of treatment (between-subject factor) and training day (within-subject factor) on escape latency. As sphericity could not be assumed based on Mauchly’s test, the Greenhouse–Geisser correction was applied to the degrees of freedom. Following a significant main effect of treatment, a post hoc analysis using Bonferroni’s correction was conducted to compare specific treatment groups at each time point. For the probe trial data, histological assessments, and gene expression data, a one-way ANOVA was used to compare groups. Tukey’s post hoc test was applied following a significant overall ANOVA result to identify specific pairwise differences between groups. The significance level of *p* < 0.05 was considered statistically significant. All statistical analyses were carried out using GraphPad Prism version 8.

## 3. Results

### 3.1. Effect of Quercetin on Spatial Learning and Memory in DEHP-Receiving Mice

**Figure 2:**
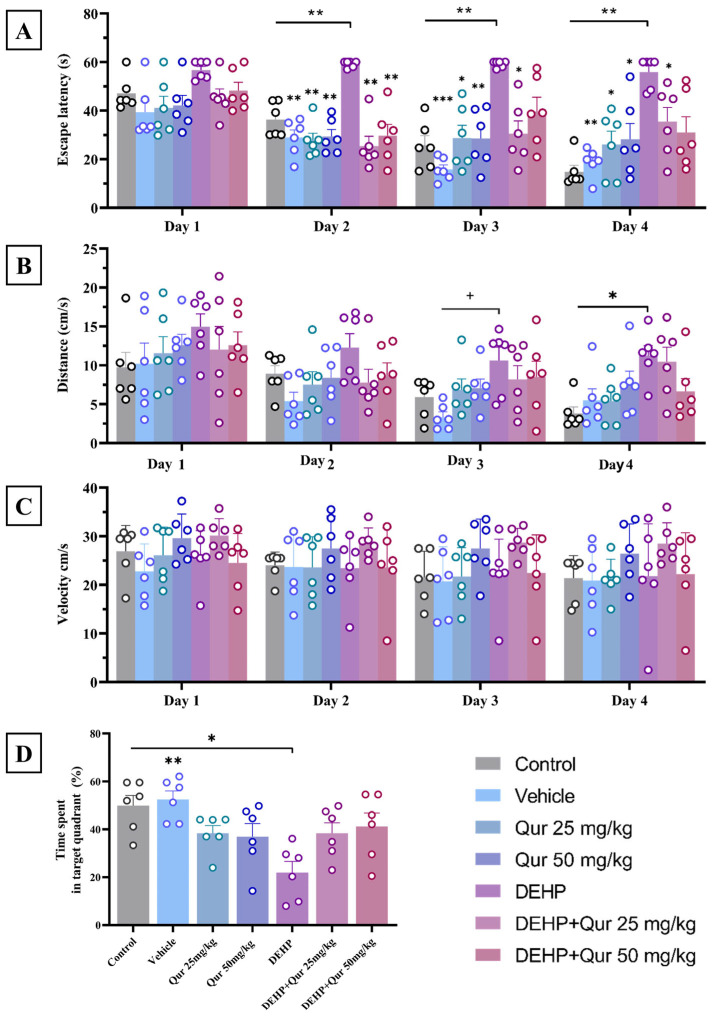
Effect of quercetin on spatial learning and memory in DEHP-treated mice. (**A**) Escape latency, (**B**) swimming distance, (**C**) swimming speed, and (**D**) time in the target quadrant. Values are expressed as mean ± SEM (n = 6 per group). * *p* < 0.05, ** *p* < 0.01, *** *p* < 0.001 vs. control; + *p* < 0.05 vs. vehicle.

The DEHP-treated group exhibited a significant increase in escape latency compared to the control group on the second (*p* = 0.0056), third (*p* = 0.0033), and fourth (*p* = 0.0024) days. Additionally, escape latency in the DEHP group was significantly higher than in the vehicle group on the second (*p* = 0.0019), third (*p* < 0.0001), and fourth (*p* = 0.0024) days. When compared to the group receiving 25 mg/kg of quercetin alone, the DEHP group also showed significantly longer escape latencies on the second (*p* = 0.0011), third (*p* = 0.0226), and fourth (*p* = 0.0468) days. A similar pattern was observed when the DEHP group was compared with the 50 mg/kg quercetin group on day 2 (*p* = 0.0017), day 3 (*p* = 0.0085), and day 4 (*p* = 0.0208). Interestingly, co-administration of quercetin at 25 mg/kg significantly improved performance in DEHP-treated mice, reducing the escape latency on days 2 (*p* = 0.0046), 3 (*p* = 0.0188), and 4 (*p* = 0.0233) compared to DEHP alone. However, the group receiving quercetin 50 mg/kg in combination with DEHP showed a statistically significant improvement only on day 2 (*p* = 0.0085), with no significant differences on the remaining later days. These results were statistically supported by repeated-measure ANOVA, which showed a significant main effect of treatment across groups (F (1.654, 8.271) = 31.76, *p* = 0.0002), a significant effect of time (F (2.838, 14.19) = 12.22, *p* = 0.0004), and a significant interaction between treatment and time (F (4.026, 20.13) = 3.047, *p* = 0.0406), indicating that the pattern of learning differed across treatment groups over time (Figure 2A).

The DEHP-treated group exhibited a significant increase in total distance traveled on the third day compared to the vehicle group (*p* = 0.0495). Throughout the testing phase, the DEHP group consistently recorded the highest average distance, while the vehicle group exhibited the shortest. Statistical analysis for swimming distance revealed a significant effect of treatment (F (1.700, 8.498) = 17.70, *p* = 0.0012), but not of time (F (3.099, 15.50) = 2.217, *p* = 0.1256), and no significant interaction effect (F (4.126, 20.63) = 0.9126, *p* = 0.4775), suggesting consistent differences across treatment groups irrespective of time (Figure 2B).

No significant differences were observed in mean swimming speed among the groups on any day. This indicates that the treatments did not affect the motor ability or swimming capacity of the animals generally. This finding was confirmed by repeated-measure ANOVA, which showed a significant main effect of treatment (F (2.135, 10.68) = 16.56, *p* = 0.0005), but no significant effect of time (F (2.674, 13.37) = 1.199, *p* = 0.3437) and no interaction effect (F (3.716, 18.58) = 0.7449, *p* = 0.5648), although there were no significant differences between groups at individual time points, as mentioned above (Figure 2C).

Mice exposed to DEHP showed a significant reduction in time spent in the target quadrant compared to both the control group (*p* = 0.0197) and the vehicle group (*p* = 0.0053), indicating impaired spatial memory recall. Additionally, the DEHP group demonstrated the lowest level of target quadrant exploration among all experimental groups, confirming a memory impairment possibly induced by DEHP exposure. In contrast, treatment with quercetin (25 and 50 mg/kg) partially improved spatial memory retention in DEHP-exposed mice. Although the time spent in the target quadrant by the DEHP + quercetin groups did not reach the levels observed in the control or vehicle groups, it was remarkably higher than that of the DEHP-only group, suggesting a neuroprotective effect of quercetin in memory recall (Figure 2D).

### 3.2. Effect of Quercetin on Neuronal Damage in the Hippocampus of DEHP-Exposed Mice

**Figure 3:**
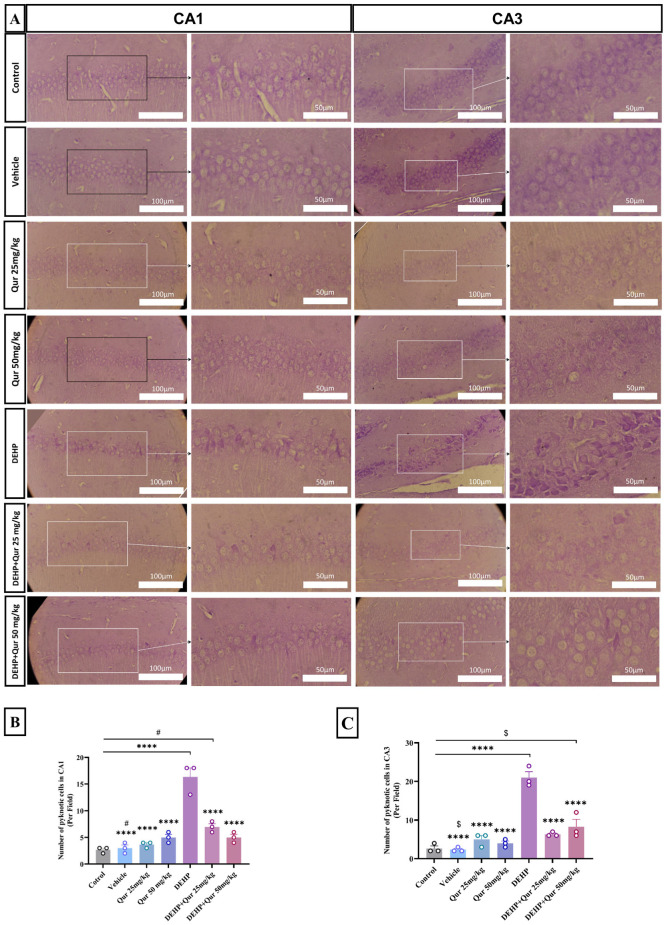
Evaluation of hippocampal pyknotic cells in DEHP-exposed mice with or without quercetin treatment. (**A**–**C**) Nissl staining of the CA1 and CA3 regions. Data are presented as mean ± SEM. Scale bar: 50 and 100 µm, n = 3. **** *p* < 0.0001 vs. control; # *p* < 0.05 DEHP + quercetin 25 mg/kg vs. vehicle; $ *p* < 0.05 DEHP + quercetin 50 mg/kg vs. vehicle.

In the CA1 sub-region, the number of damaged neurons was significantly higher in the DEHP group compared to the control, vehicle, and quercetin-only groups (25 and 50 mg/kg) (*p* < 0.0001). Co-administration of quercetin with DEHP at both 25 mg/kg and 50 mg/kg significantly reduced the number of damaged neurons compared to the DEHP group (*p* < 0.0001). However, despite this reduction, the number of injured neurons in the DEHP + quercetin 25 mg/kg group remained significantly higher than in the control (*p* < 0.0001), vehicle (*p* = 0.0362), and quercetin 25 mg/kg groups (*p* < 0.0001). Similarly, the DEHP + quercetin 50 mg/kg group also showed a higher number of injured cells compared to the control (*p* < 0.0001). Moreover, the DEHP + quercetin 25 mg/kg group exhibited significantly more neuronal damage than the DEHP + quercetin 50 mg/kg group, suggesting a dose-dependent protective effect (F (6, 14) = 36.42, *p* < 0.0001) (Figure 3A,B).

In the CA3 region, DEHP exposure led to a marked increase in neuronal damage compared to the vehicle, control, and both quercetin-alone groups (*p* < 0.0001). Treatment with quercetin at 25 mg/kg or 50 mg/kg significantly reduced neuronal injury compared to the DEHP group (*p* < 0.0001). Furthermore, the DEHP + quercetin 50 mg/kg group demonstrated damaged neurons compared to the control and vehicle groups (*p* = 0.0249 and *p* = 0.0166, respectively), though no significant difference was observed when compared to the quercetin-only 50 mg/kg group (*p* = 0.1205) (F (6, 14) = 37.76, *p* < 0.0001) (Figure 3A,C).

### 3.3. Histomorphometric Evaluation of Hippocampal Neurons Following Quercetin Treatment in DEHP-Exposed Mice

**Figure 4:**
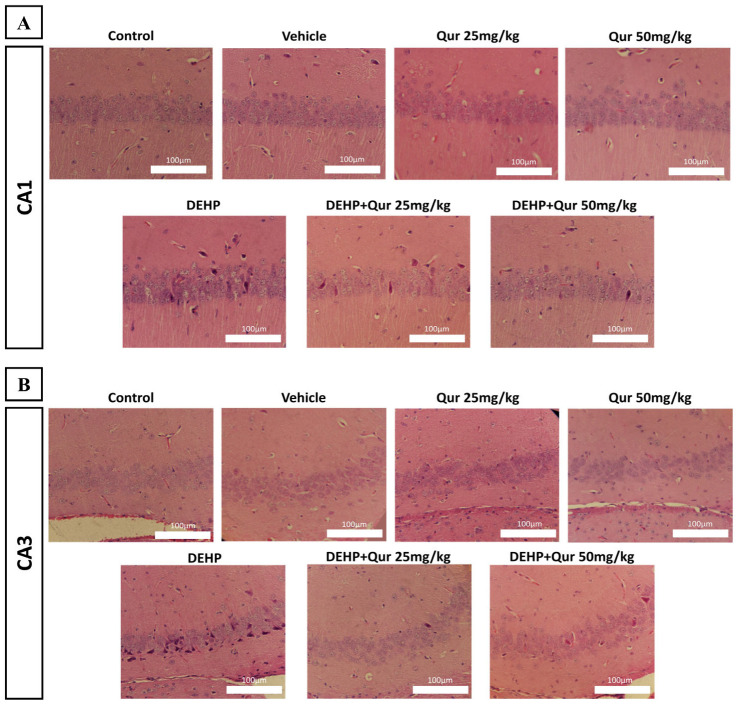
Histomorphometric evaluation of hippocampal neurons in DEHP-exposed mice with or without quercetin treatment. H&E and Nissl staining of the CA1 and CA3 regions revealed marked neuronal disorganization, pyknosis, and necrosis in DEHP-treated mice, while quercetin co-treatment preserved neuronal morphology and reduced neuronal damage. (**A**,**B**) H&E staining. Scale bar: 100 µm, n = 3.

To investigate the histopathological changes in the hippocampus, H&E staining was performed on the CA1 and CA3 sub-regions of the hippocampus. In the control group, pyramidal neurons appeared normal due to their structure, with a uniform arrangement of cell layers and hardly stained nuclei, which indicates healthy morphology. In contrast, tissue sections from the DEHP-treated group revealed marked neuronal disorganization and degenerative changes. The alignment of cells was irregular, and the intensity of affected neurons was notably increased compared to the control. On the other hand, histological evaluation of the treatment groups indicated that quercetin modulated DEHP-induced damage. In the DEHP + quercetin (50 mg/kg) group, the CA1 region demonstrated notable structural preservation, with improved organization and fewer necrotic neurons. In the DEHP + quercetin (25 mg/kg) group, the CA3 region exhibited the most observed histological improvement, including more organized cell layers and reduced signs of neuronal damage (Figure 4A,B).

### 3.4. Quercetin Attenuates DEHP-Induced Astrocyte Activation in the Hippocampus

**Figure 5:**
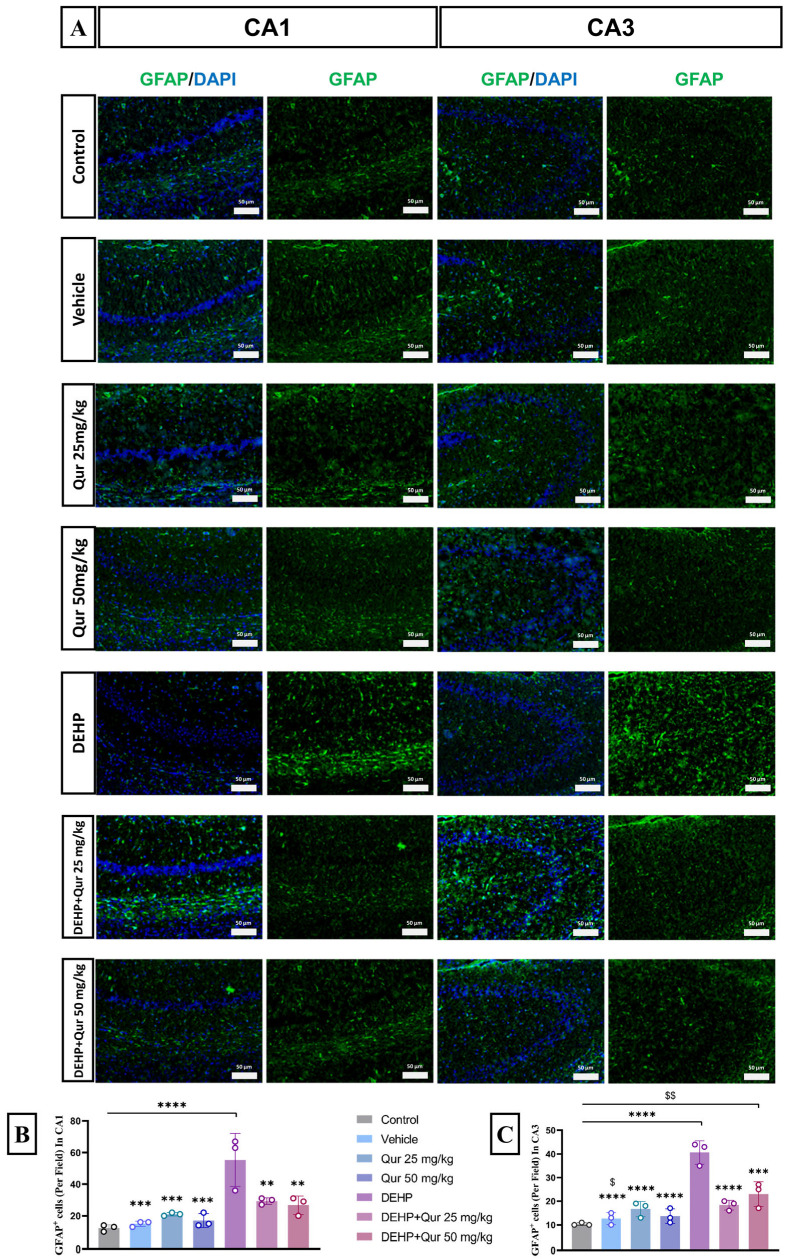
Quercetin attenuates DEHP-induced astrocyte activation in the hippocampus. (**A**–**C**) GFAP immunofluorescence in CA1 and CA3 regions. ** *p* < 0.01, *** *p* < 0.001, **** *p* < 0.0001 vs. control; ^$^ *p* < 0.05, ^$$^ *p* < 0.001 vs. DEHP + quercetin 50 mg/kg. Scale bar: 50 µm, n = 3.

To assess astrocyte reactivity in the hippocampus, GFAP immunofluorescence staining was performed in the CA1 and CA3 sub-regions (Figure 5A).

In the CA1 region, quantitative analysis revealed a significant increase in GFAP-positive cells in the DEHP-treated group compared to the control (*p* < 0.0001), vehicle (*p* = 0.0001), and quercetin 50 mg/kg (*p* = 0.0002) groups (F (6, 14) = 12.80, *p* < 0.0001) (Figure 5B).

In the CA3 region, DEHP exposure also resulted in a marked increase in GFAP-positive cells relative to the control, vehicle, quercetin 25 mg/kg, and quercetin 50 mg/kg groups (*p* < 0.0001). Notably, the group treated with quercetin 50 mg/kg + DEHP also exhibited a significantly higher GFAP expression than the control (*p* = 0.0069), suggesting that while quercetin mitigated DEHP-induced astrocyte reactivity, it did not completely restore it to baseline (Figure 5C).

### 3.5. Quercetin Attenuates DEHP-Induced Upregulation of Proinflammatory Cytokines

**Figure 6:**
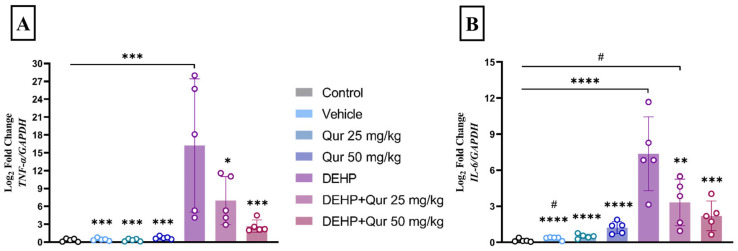
Quercetin reduces DEHP-induced proinflammatory cytokine expression in the hippocampus. (**A**,**B**) qRT-PCR revealed that DEHP upregulated *TNF-α* and *IL-6* expression, and quercetin significantly reduced both cytokines in a dose-dependent manner. * *p* < 0.05, ** *p* < 0.01, *** *p* < 0.001, **** *p* < 0.0001 vs. control; # *p* < 0.05 vs. DEHP + quercetin 25 mg/kg. Values represent mean ± SEM (n = 5).

The DEHP group showed a marked increase in *TNF-α* mRNA levels compared to control, vehicle, quercetin 25 mg/kg, and quercetin 50 mg/kg groups (*p* < 0.0001). Administration of quercetin 50 mg/kg + DEHP significantly attenuated *TNF-α* expression when compared to DEHP alone (*p* = 0.0003). The 25 mg/kg dose also did reach statistical significance (*p* = 0.0153). (F (6, 28) = 8.649, *p* < 0.0001) (Figure 6A).

Analysis of *IL-6* gene expression revealed a significant increase in the DEHP-treated group compared to the control, vehicle, quercetin 25 mg/kg, and quercetin 50 mg/kg groups (*p* < 0.0001). Importantly, co-treatment with quercetin significantly reduced *IL-6* expression compared to DEHP alone at both 25 mg/kg (*p* = 0.0009) and 50 mg/kg (*p* < 0.0001) doses (F (6, 28) = 15.24, *p* < 0.0001) (Figure 6B).

## 4. Discussion

The present study showed that DEHP exposure significantly impaired spatial learning and memory performance in mice, as exhibited by increased escape latency, longer swimming distances, and reduced target quadrant preference in the MWM test. These behavioral impairments were contributed with marked histopathological changes in hippocampal CA1 and CA3 neurons, increased astrocytic activation, and upregulation in *IL-6* and *TNF-α*. Importantly, co-administration of quercetin mitigated these adverse effects in a dose-dependent manner. Quercetin treatment improved cognitive performance, preserved hippocampal neuronal structure, inhibited astrocytic reactivity, and suppressed neuroinflammatory cytokine expression, indicating its potential neuroprotective role against DEHP-induced cognitive and neuronal damage.

The hippocampus is essential for both learning and memory by modulations related to plastic synaptic connections between neurons of this region. To assess cognitive functions, we used the MWM to compare spatial learning and memory across groups. Mice exposed to DEHP needed significantly more time and swimming distance to locate the hidden platform during training compared to control or vehicle groups, indicating slower habitation of the task. On the probe trial following the platform removal, the DEHP group spent a markedly shorter percentage of time in the target quadrant, reflecting impaired spatial memory preservation. These behavioral impairments reflect findings similar in the literature such as anxiety-like behavior production and working memory impairment in multigenerational DEHP exposure in rats [27,28]. Thus, our results confirm that DEHP exposure negatively affects hippocampus-dependent learning and memory procedures.

Long-term potentiation (LTP) at hippocampal synapses underlies learning and memory. In LTP, high-frequency stimulation causes NMDA receptor opening and Ca^2+^ influx, leading to entry of AMPA receptors at the post-synaptic membrane and strengthening the synapse. Impairment of NMDA/AMPA signaling or inhibition of these receptors in an extensive manner impairs LTP and furthermore cognition [29]. DEHP is known to interfere with synaptic plasticity. It can reduce the expression of NMDA receptor subunits (NR1, NR2) and inhibit receptor functioning in hippocampal neurons, which may have caused the observed deficits in spatial learning and memory [9,11]. Additionally, DEHP’s negative endocrine effects (e.g., through thyroid hormone dysregulation or PPAR activation) can delay neurodevelopment and cognitive maturation. For example, DEHP exposure has been associated with reduced testosterone levels in developmental phase of males, which may eliminate the hormone’s neuroprotective impact and contribute to synaptic deficits and memory impairment. In summary, DEHP induces hippocampal dysfunction through multiple varied mechanisms including oxidative stress, inflammation, and endocrine dysfunction [11,27].

In contrast, quercetin-treated mice showed marked improvements in learning and memory function. Notably, quercetin at 50 mg/kg exhibited better performance than 25 mg/kg on the probe trial, indicating more efficient memory preservation, even though during the initial days the 25 mg/kg dose appeared to induce early learning. This suggests a dose-dependent effect of quercetin on different phases of memory procedures. While the exact mechanisms were not straightforwardly measured here, previous studies show that quercetin can enhance cognitive function through multiple pathways. For example, quercetin significantly improved the MWM performance and reduced hippocampal neuronal damage in an Alzheimer’s disease mouse model [2,30]. These improvements were accompanied by increased levels of intrinsic antioxidants (glutathione, superoxide dismutase, and catalase) and reduced markers of oxidative stress, indicating activation of the Nrf2/HO-1 antioxidant pathway [31,32]. Indeed, quercetin’s neuroprotective effects largely contributed with its upregulation of Nrf2-mediated antioxidant protections and inhibition of apoptosis. In our study, quercetin treatment likely modulated hippocampal response to DEHP by similar mechanisms, as evidenced by improved behavioral outcomes.

Different outcomes for 25 vs. 50 mg/kg quercetin suggest a complicated dose-dependent response. Apparently, the lower dose may have supported initial encoding of the spatial memory, while the higher dose more strongly established the memory trace for retention on the probe day. The biphasic response may mirror distinct signaling plateau in hippocampal-responsible mechanisms. Moreover, when quercetin was given with the absence of DEHP, the memory effects differed, indicating that DEHP exposure may affect quercetin’s either pharmacokinetics or pharmacodynamics. DEHP and quercetin may interact to change absorption, metabolism or blood–brain compatibility of quercetin. In addition, DEHP itself could modulate these molecular targets (e.g., receptors, transcription factors) so that quercetin’s protective pathways are either enhanced or worsened in its presence. Investigating whether DEHP potentiates or interrupts with quercetin’s antioxidant and anti-inflammatory properties will necessitate further mechanistic studies. Whatsoever, these findings indicate the probable complicated network of quercetin–DEHP interactions in the hippocampus region [28,33].

Histological examination of the hippocampus had provided further insight. In DEHP-exposed mice, H&E and Nissl staining revealed a significant increase in dark, shrunken pyramidal neurons within CA1 and CA3, along with disarranged cellular organization. Such neuronal damage likely explains the behavioral dysfunctions, since CA1 and CA3 networks are essential for spatial mapping and pattern recognition. Importantly, previous studies have shown that DEHP exposure causes neuro-architectural damage and neuronal apoptosis [11,34], including activation of the mitochondrial (cytochrome c–caspase) apoptosis pathway. More aligned, we observed elevated markers of apoptosis and inflammation (e.g., higher caspase-3/Bax and lower Bcl-2/AKT levels, as reported elsewhere [11]) in DEHP brains. Thus, DEHP appears to induce significant hippocampal pathology, contributing to the observed learning and memory impairments.

Quercetin treatment substantially mitigated the histopathology. Both quercetin + DEHP groups (25 and 50 mg/kg) showed significantly fewer dark neurons in CA1 and CA3 compared to DEHP alone. In other words, quercetin preserved normal pyramidal neuron morphology despite DEHP exposure. This neuroprotection is aligned with many reports of quercetin’s advantages on brain tissue. Quercetin is known to potentially affect as an anti-inflammatory and antioxidant factor in the CNS [35]. It scavenges ROS, upregulates antioxidants endogenously, and inhibits pro-degenerative signaling. For example, quercetin has been shown to reverse LPS-induced neuronal degeneration and preserve cognitive function in rodent hippocampus [36]. It can also reduce lipid peroxidation and inflammatory factors, thereby preventing cellular loss and death [16]. In our study, characteristics such as quercetin’s antioxidant activity likely underlie the reduced neuronal damage, while its anti-inflammatory signaling (e.g., via Nrf2 and inhibition of NF-κB) would suppress pro-apoptotic pathways [37,38].

Interestingly, the two quercetin doses had differential regional effects. In the CA3 region, quercetin at 25 mg/kg with DEHP reduced dark neurons more than the 50 mg/kg dose. In contrast, in CA1 the 50 mg/kg dose showed greater enhancement. This pattern suggests that CA1 and CA3 respond differently to quercetin in various dosage. CA3 is known for its dense recurrent excitatory collaterals (the “auto-associative” network) that underlie pattern completion, whereas CA1 receives processed input and is essential for encoding spatial information [39]. It may be that CA3 neurons benefit more from the moderate quercetin dose during initial learning, while CA1 neurons require stronger antioxidant protection during memory consolidation. Alternatively, differences in local metabolism or in the BBB at these sub-regions might affect the quercetin concentration in its efficient way [40]. In any case, these findings highlight the functional and pharmacological heterogeneity within the hippocampus and suggest further study of subfield-specific mechanisms of quercetin action.

Astrocytes play critical roles supporting neurons, synaptic function and neuro-immunologic reactions. Under traumatic conditions like DEHP exposure, astrocytes reactivate and can amplify neuroinflammation by cytokines and chemokines release [41,42]. We found that DEHP markedly increased GFAP immune reactivity in the hippocampus, indicating the astrocyte activation. In the quercetin-treated groups, astrocyte activity was significantly lower than in DEHP alone, though it was still measured above control levels. This indicates that quercetin incompletely prohibited block the astrocytic response. In particular, quercetin (both doses) reduced astrocyte activation in CA1 and CA3 relative to DEHP. Quercetin’s anti-inflammatory action likely underlies this effect by inhibiting NF-κB and other inflammatory pathways; quercetin would reduce the signals that drive astrocytes into a reactive phase. Indeed, nano-liposomal quercetin has been shown to shift glial cells from a pro-inflammatory to an anti-inflammatory phenotype in a neurodegenerative model [33].

Repeatedly, we noted mentioned regional differences. In CA3, the lower quercetin dose (25 mg/kg) produced a greater decrease in astrocyte activity than the higher dose, whereas in CA1 the 50 mg/kg dose was shown to be more efficient. This could reflect our neuronal findings and suggest a consistent pattern of regional dose sensitivity and specificity. Astrocytes in different hippocampal subfields may express different receptor regulation or metabolic enzymes, leading to these patterns. Overall, quercetin’s antioxidant and anti-inflammatory properties seem to differentially modulate astrocyte dynamics in CA1 vs. CA3, further noting the complexity of its way of action in the hippocampus [43,44,45].

Since reactive astrocytes are a major leading cause of pro-inflammatory cytokines release, we also measured gene expression of IL-6 and TNF-α in the hippocampus region [39]. DEHP exposure significantly upregulated both IL-6 and TNF-α transcription compared to control, confirming a strong pro-inflammatory response. Quercetin co-treatment at both administered doses significantly reduced IL-6 and TNF-α expression vs. DEHP alone, consistent with quercetin’s known anti-cytokine effects. In microglia and astrocytes, quercetin can inhibit iNOS and NF-κB, leading to lower production of TNF-α, IL-1β and other inflammatory mediators. Based on our results, the suppression of IL-6 and TNF-α likely reflects these anti-inflammatory pathways. For example, quercetin has been reported to modulate MAPK and NF-κB signaling, which dampens cytokine transcription [46,47]. The mitigation of inflammation by quercetin would lead to neuron protection from cytokine-induced oxidative damage. Eventually, quercetin’s ability to reduce DEHP-induced neuroinflammation, lowering glial activation and cytokine levels, likely contributes to its overall neuroprotective effect observed in our study [48].

Despite our findings, several limitations of this study should be pointed out. First, we merely focused on astrocytic activation and did not examine the role of microglia, which are also known to be key regulators of neuroinflammation and could significantly contribute to DEHP-induced neurotoxicity. On the other hand, we measured only IL-6 and TNF-α as representative pro-inflammatory cytokines; evaluation of additional inflammatory mediators (e.g., IL-1β, iNOS, and COX-2) would provide a more overall picture of the inflammation-related pathways. Also, this study did not measure oxidative stress index in brain tissue and the proposed antioxidant cooperation is based on the previously published literature. Although our results suggest dose-dependent effects of quercetin, only two dosages were tested, and a wider dose range with pharmacokinetic profiling would help to clarify the optimal therapeutic window. Finally, this was an acute experimental model evaluating merely adult male mice, hence long-term outcomes of DEHP exposure and quercetin treatment along with examining mentioned alterations in female mice remain unexplored in the present investigation. Future studies should include chronic exposure duration, assessment of microglial activity, wider markers of inflammatory and oxidative markers, and mechanistic approaches such as receptor blockade or genetic models to validate the pathways involved. These investigations will further exhibit the neuroprotective mechanisms of quercetin and prove its potential as a protection against environmental neuro-toxicants like DEHP.

## 5. Conclusions

In conclusion, our data indicate that quercetin, particularly at the dose of 50 mg/kg, effectively mitigated the adverse effects of DEHP on the hippocampus. Quercetin reduced DEHP-induced upregulation of inflammatory genes, preserved neuronal structure, improved performance in learning and memory tasks, and lowered astrocyte reactivity. These findings suggest that quercetin may have therapeutic potential to mitigate DEHP neurotoxicity. Future studies should investigate the optimal dosing regimen and duration of quercetin, as well as its effects in various exposure models.

## Figures and Tables

**Table 1:** The primer sequences.

Gene	Forward (5′-3′)	Reverse (5′-3′)
*TNF-α*	CAGGCGGTGCCTATGTCTC	CGATCACCCCGAAGTTCAGTAG
*IL-6*	GAGGATACCACTCCCAACAGACC	AAGTGCATCATCGTTGTTCATACA
*GAPDH*	AGGTCGGTGTGAACGGATTTG	TGTAGACCATGTAGTTGAGGTCA

## Data Availability

The raw data supporting the conclusions of this article will be made available by the authors upon reasonable request. The data are not publicly available due to specific ethical and privacy considerations.

## Abbreviations

The following abbreviations are used in this manuscript:

PVC	Polyvinyl chloride
DEHP	Di(2-ethylhexyl) phthalate
BBB	Blood–brain barrier
TNF-α	Tumor necrosis factor-alpha
IL-6	Interleukin-6
ROS	Reactive oxygen species
Qur	Quercetin
MWM	Morris water maze
qRT-PCR	Quantitative real-time polymerase chain reaction
H&E	Hematoxylin–eosin
OCT	Optimal cutting temperature
PBS	Phosphate-buffered saline
PFA	Paraformaldehyde
ROI	Region of Interest
DAPI	4′,6-Diamidino-2-phenylindole
cDNA	Complementary DNA
SEM	Standard error of the mean
ANOVA	Analysis of variance
EDC	Endocrine-disrupting chemical
LTP	Long-term potentiation
NR	NMDA receptor
